# Variants in *CCL16* are associated with blood plasma and cerebrospinal fluid CCL16 protein levels

**DOI:** 10.1186/s12864-016-2788-x

**Published:** 2016-06-29

**Authors:** Mark T. W. Ebbert, Lyndsay A. Staley, Joshua Parker, Sheradyn Parker, Matthew Bailey, for the Alzheimer’s Disease Neuroimaging Initiative, Perry G. Ridge, Alison M. Goate, John S. K. Kauwe

**Affiliations:** Department of Biology, Brigham Young University, Provo, UT 84602 USA; Biology and Biomedical Sciences, Washington University, St. Louis, MO 63110 USA; Department of Neuroscience Icahn School of Medicine, New York, NY 10029 USA

**Keywords:** Blood, Brain, CCL16, Plasma, Cerebrospinal fluid, Genetics, Association

## Abstract

**Background:**

*CCL16* is a chemokine predominantly expressed in the liver, but is also found in the blood and brain, and is known to play important roles in immune response and angiogenesis. Little is known about the gene’s regulation.

**Methods:**

Here, we test for potential causal SNPs that affect CCL16 protein levels in both blood plasma and cerebrospinal fluid in a genome-wide association study across two datasets. We then use METAL to performed meta-analyses with a significance threshold of *p* < 5x10^−8^. We removed SNPs where the direction of the effect was different between the two datasets.

**Results:**

We identify 10 SNPs associated with increased CCL16 protein levels in both biological fluids.

**Conclusions:**

Our results will help understand *CCL16*’s regulation, allowing researchers to better understand the gene’s effects on human health.

**Electronic supplementary material:**

The online version of this article (doi:10.1186/s12864-016-2788-x) contains supplementary material, which is available to authorized users.

## Background

Chemokines are specialized cytokines (cell-signaling proteins) that induce chemotaxis in proximal cells. The CC subfamily is primarily a chemoattractant to monocytes and lymphocytes [1], demonstrating their role in immune response. Chemokines are highly conserved across species, suggesting their roles are essential to viable offspring [2]. They have been implicated specifically in brain health, including neuronal migration during development and even neuronal death [2, 3], which has important implication on brain health throughout life and could have important implications in neurodegenerative diseases. *CCL16* is part of the CC chemokine subfamily and is predominantly expressed in the liver [4]. The CCL16 protein is also known to be present and active in the brain and blood [3, 5].

*CCL16* is located on the q arm of chromosome 17 amongst a cluster of other chemokines and is known to play a role in angiogenesis [5]. While the CCL16 protein is known to be heavily active in the liver, little is known about its overall regulation in the brain and blood.

To date, most research regarding *CCL16* is limited to its expression in the liver and its role in chemotaxis, generally. Little is known about *CCL16* gene regulation and the protein’s role across all tissues. It is clearly involved in immune response, as one of its primary functions is to attract lymphocytes and monocytes [1], making *CCL16* a potentially critical protein throughout the body. It is also unclear how CCL16 protein levels across blood plasma and CSF are regulated.

Here, we measure CCL16 protein levels in cerebrospinal fluid (CSF) and blood plasma, and perform a genome-wide association analysis to identify SNPs that are associated with CCL16 levels in both CSF and blood plasma. These findings will help clarify *CCL16* regulatory mechanisms and their effects on human development and health.

## Methods

### Subjects and data description

Exactly 246 and 240 CSF and blood plasma samples, respectively, were used in this study from participants in the Knight-Alzheimer’s disease Research Center at Washington University School of Medicine (Knight ADRC) and 297 and 347 CSF and blood plasma samples, respectively, were used from the Alzheimer’s Disease Neuroimaging Initiative (ADNI). From the Knight ADRC samples, approximately 93 % of the samples were controls, and 7 % were Alzheimer’s disease cases, and from the ADNI samples, approximately 85 % were controls, and 15 % were AD cases. We measured levels for CCL16 in each sample using the Human DiscoveryMAP Panel v1.0 and a Luminex 100 platform [6]. All samples were genotyped using the Illumina 610 or the Omniexpress chip. The Knight ADRC samples and associated collection methods were previously described [7, 8]. We collected the ADNI samples from the ADNI database (adni.loni.usc.edu), which were part of the ADNI biomarker study [9]. All samples are of European descent.

### SNP imputation

The SNPs were imputed as previously described [6]. Briefly, data from the 1000 Genomes Project (June 2012 release) were used to impute SNPs using Beagle. Imputed SNPs with the following criteria were removed: (1) an r^2^ of 0.3 or lower, (2) a minor allele frequency (MAF) lower than 0.05 (3) out of Hardy-Weinberg equilibrium (*p* < 1 × 10 − 6), (4) a call rate lower than 95 %, or (5) a Gprobs score lower than 0.90. Exactly 5,815,690 SNPs passed the QC process.

### Data cleaning and analysis

We excluded SNPs that exceeded thresholds for Hardy-Weinberg Equilibrium [10, 11] (−−hwe 0.00001), missing genotype rate (−−geno 0.05), and minor allele frequency (−−maf 0.01) for each data set, using PLINK version 1.07 [12], to perform genotype quality control. We then excluded individuals with a missing genotyping rate greater than 2 % (−−mind 0.02), leaving 246 individuals from Knight ADRC and 282 samples from ADNI after cleaning. Remaining Knight ADRC and ADNI samples consisted of 40 and 61 % males, respectively. The average age for ADNI samples was 76 years, ranging from 58 to 91 years, and the average age for Knight ADRC samples was 73, ranging from 49 to 91.

After data cleaning, we tested for an association between each remaining SNP and CCL16 CSF levels within each dataset, adjusting for age, gender, and the first two principal components generated by EigenSoft [13, 14]. We then performed a meta-analysis across both data sets, accounting for sample size, p-values, and direction of effect using the default METAL [15] settings. We retained all SNPs that had a genome-wide significant meta-analysis p-value less than 5×10^−8^ and that had the same direction of effect in both datasets. We then tested associations between each SNP and CCL16 plasma levels following the same protocol and kept only those SNPs that were significantly associated with both CSF and plasma CCL16 levels.

To assess known functional effects and identify SNPs that are biologically likely to modify gene expression or function, we searched all significant SNPs in the NHGRI catalog of published genome-wide association studies [16] (downloaded July, 2015) for known disease associations, collected RegulomeDB annotations (accessed September, 2015) [17], and collected functional annotations from wANNOVAR [18, 19].

We performed a conditional analysis for all included SNPs that were in or near a given region to test whether there is one or multiple independent effects in the region [20]. Conditional analysis is a follow-up method used to test if there are secondary association signals within a region by retesting each SNP while including the top SNP as a covariate. We chose the most significant SNP in the region to use as a covariate in the conditional analysis.

## Results

We identified 34 and 25 SNPs significantly associated with CCL16 protein levels in CSF and plasma, respectively (Additional file 1 and Additional file 2). Of these, 10 SNPs were significantly associated with increased CCL16 protein levels in both CSF and plasma, based on the meta-analyses, all of which are in or near the CCL16 gene (Table 1). The genomic inflation factor was 1.0 (q-q plots can be found in Additional file 3 and Additional file 4). None of the 10 SNPs have been reported to show association with disease in the NHGRI GWAS catalog. Two SNPs are located in the 3’ untranslated region (UTR), 4 are intronic, 1 is downstream, and 3 are intergenic (Table 1). SNPs are identified as “downstream” if they are within 500 nucleotides of the 3’ end of a gene, according to the National Center for Biotechnology Information’s (NCBI) SNP FAQ Archive [21]. Minor allele frequencies (MAF) for the ten SNPs ranged from 0.06 to 0.14 and RegulomeDB scores ranged from ‘1f’ to ‘6’, with three SNPs having no known regulation data, according to RegulomeDB.

**Table 1:** Significant SNPs in or near the CCL16 gene on chromosome 17 that met our inclusion criteria with pertinent biological information implicating them in CCL16 regulation

SNP	Base Pair position	Minor Allele	Major Allele	MAF	Predicted Function	RegulomeDB score	Meta-analysis p-value	
							CSF	Plasma
rs80329614	34303312	C	T	0.1406	downstream	3a	1.666E-19	5.853E-28
rs11080368	34305071	A	C	0.1040	intronic	No data	2.321E-19	1.777E-27
rs11080369	34305164	C	A	0.1042	intronic	1f	2.321E-19	1.777E-27
rs33995560	34303771	C	T	0.1122	UTR3	No data	2.941E-19	2.094E-27
rs7216969	34305048	A	G	0.1040	intronic	6	2.941E-19	2.094E-27
rs150951362	34304264	A	G	0.1008	UTR3	6	1.65E-17	2.05E-20
rs75236781	34306470	C	G	0.0613	intronic	6	2.958E-17	3.942E-20
rs149197550	34295254	T	C	0.0641	intergenic	No data	8.32E-16	9.20E-18
rs4795104	34287400	T	A	0.0639	intergenic	6	9.626E-16	1.015E-17
rs4796144	34293003	A	G	0.0641	intergenic	6	9.626E-16	1.015E-17

**Fig. 1:**
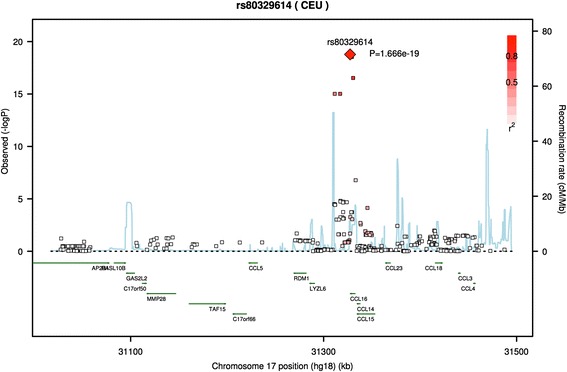
SNPs located in and around CCL16 are associated with CCL16 protein levels in cerebrospinal fluid (CSF). SNP rs80329614 is the SNP most strongly associated with CCL16 protein levels in CSF. All significant SNPs are in high linkage disequilibrium, suggesting there is only one association signal in the region

**Fig. 2:**
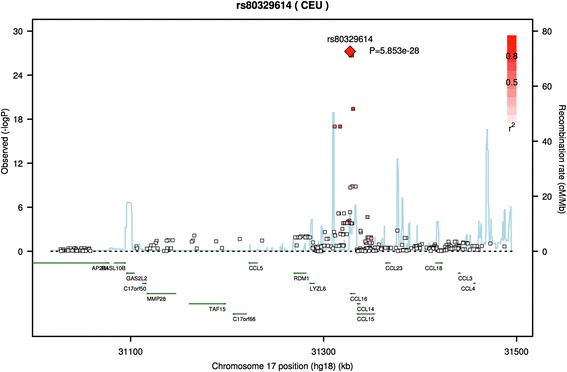
SNPs located in and around the CCL16 gene are associated with CCL16 protein levels in blood plasma. As in the CSF analysis, rs80329614 is the SNP most strongly associated with CCL16 protein levels in plasma. All significant SNPs are in high linkage disequilibrium, suggesting there is only one association signal in the region

All ten SNPs associated with both CSF and plasma CCL16 protein levels are in high linkage disequilibrium, suggesting there is likely one association signal in the region (Figs. 1 and 2). The conditional analysis further supports a single signal as the p-values are no longer genome-wide significant when including the most significant SNP, rs80329614, as a covariate. SNP rs80329614 is the SNP identified as being “downstream” of *CCL16* (Table 1, Figs. 1 and 2) and has a RegulomeDB score of ‘3a’.

SNP rs11080369 is an intronic SNP with a RegulomeDB score of ‘1f’ and was previously demonstrated to be within an expression quantitative trait locus (eQTL) that specifically modifies CCL16 gene expression [22]. The remaining 8 SNPs do not have known regulatory associations or functions, according to RegulomeDB, but 5 of the 8 are located in genic regions, such as UTRs and introns.

## Discussion

We identified exactly 10 SNPs associated with increased CCL16 protein levels in both CSF and blood plasma, all of which were located in or around the *CCL16* gene and based on the conditional analysis, all representing on single signal. The SNP most strongly associated with CCL16 protein levels was rs80329614, which was identified as being “downstream” (within 500 nucleotides downstream) of *CCL16* with a RegulomeDB score of “3a”. RegulomeDB scores range from “1a” to “6” where lower scores indicate stronger evidence that the SNP affects gene regulation based on both empirical data, such as ChIP-seq, and whether the SNP is within a known transcription factor binding motif. A score of “3a” indicates minimal evidence that a SNP is involved in gene regulations, but RegulomeDB can only represent what is currently known based on experimental data. Thus, further investigation of this SNP may be warranted. SNP rs11080369 received a score of “1f”, indicating that it is known to be part of an eQTL. As such, the rest of the SNPs are likely part of the same eQTL since they are all in strong linkage disequilibrium.

Two of the remaining SNPs (rs33995560 and rs150951362) are located in the 3’UTR of *CCL16*, which can play an important role in gene transcription and translation [23–25], while three others (rs11080368, rs7216969, rs75236781) are located within *CCL16* introns, which can also affect gene regulation [25]. The remaining three SNPs (rs149197550, rs4795104, and rs4796144) are located between approximately 8000 and 16000 nucleotides downstream of the gene. Intergenic variants are generally less likely to affect transcription than variants within the promoter region or the gene itself, though it is possible if the variant affects transcription factor binding. Many genes have enhancers both upstream and downstream that can be active in specific tissues, depending on the transcription factors expressed in the tissue [26, 27]. However, given that rs149197550, rs4795104, and rs4796144 are in high linkage disequilibrium with the other significant SNPs, we believe they are less likely to be causal variants. The remaining three are intergenic. Identifying which SNP(s) directly affect *CCL16* regulation will require experimental data, but we believe the most suspect from these 10 is rs80329614 because it has the strongest association, its proximity to the gene, and the fact that many genes have regulatory elements (e.g., enhancers) downstream [26, 27], though the VISTA enhancer database does not have data on *CCL16* enhancers [26].

While these 10 SNPs are the most significant and biologically likely to affect *CCL16* regulation based on our criteria, there may be other SNPs in the individual CSF and plasma lists that regulate *CCL16* independently, including those found in other genes. More biological data will be necessary to identify causal SNPs. Additionally, our data are not whole exome or genome and there may be causal variants in LD with our top hits, associated with the single signal we’ve seen in our results that were not yet genotyped. Full sequencing data within the region may reveal other candidate causal variations.

## Conclusions

Our results show that one or more SNPs in or around the *CCL16* gene are associated with increased CCL16 protein levels in both CSF and plasma, but it is not clear whether that is due to regulation in multiple tissues such as the brain and liver, or whether the protein is crossing the blood–brain barrier. Our results are an important step to resolving this relationship, as they will allow researcher to explore experimentally how these SNPs affect *CCL16* gene regulation in different tissues. Understanding *CCL16* gene regulation in greater depth will be important to better understanding its role in human health.

## Abbreviations

ADNI, Alzheimer’s Disease Neuroimaging Initiative; CSF, Cerebrospinal Fluid; eQTL, expression quantitative trait locus; Knight ADRC, Knight-Alzheimer’s Disease Research Center at Washington University School of Medicine; LD, Linkage Disequilibrium; SNP, single nucleotide polymorphism; UTRs, untranslated regions

## Additional files

Additional file 1: File contains a table of SNPs significantly associated with CCL16 levels in blood plasma by meta-analysis. (DOCX 88 kb) Additional file 2: File contains a table of SNPs significantly associated with CCL16 levels in CSF by meta-analysis. (DOCX 94 kb) Additional file 3: File contains a Q-Q plot of the CSF data used in this study. (DOCX 82 kb) Additional file 4: File contains a Q-Q plot of the plasma data used in this study. (DOCX 83 kb)
