# Supplementary material for: Variants in CCL16 are associated with blood plasma and cerebrospinal fluid CCL16 protein levels
Source: BMC Genomics. 2016 Jun 29;17(Suppl 3):437. doi: 10.1186/s12864-016-2788-x (PMC4943476; doi:10.1186/s12864-016-2788-x)
Supplement: Additional file 1: — File contains a table of SNPs significantly associated with CCL16 levels in blood plasma by meta-analysis. (DOCX 88 kb) [file 12864_2016_2788_MOESM1_ESM.docx]

| **SNP** | **Chromosome** | **Chromosomal position** | | **Proximal Gene(s)** | | **MAF** | | **Predicted Function** | | **Meta-analysis**  **p-value** | |
| --- | --- | --- | --- | --- | --- | --- | --- | --- | --- | --- | --- |
| rs9877182 | 3 | 23323465 | UBE2E2 | | 0.082 | | intronic | | 5.43E-09 | |  |
| rs113882198 | 5 | 1691032 | LOC728613,MIR4277 | | 0.048 | | intergenic | | 4.22E-09 | |  |
| rs1875196 | 5 | 1691202 | LOC728613,MIR4277 | | 0.048 | | intergenic | | 4.22E-09 | |  |
| rs184623326 | 5 | 1694882 | LOC728613,MIR4277 | | 0.021 | | intergenic | | 1.47E-09 | |  |
| rs9483551 | 6 | 133480032 | LINC00326,EYA4 | | 0.049 | | intergenic | | 5.57E-09 | |  |
| rs75943876 | 6 | 133484317 | LINC00326,EYA4 | | 0.04 | | intergenic | | 5.78E-09 | |  |
| rs74612956 | 8 | 9787551 | MIR124-1,MSRA | | 0.083 | | intergenic | | 1.79E-08 | |  |
| rs12004619 | 9 | 22365565 | CDKN2B-AS1,DMRTA1 | | 0.089 | | intergenic | | 2.16E-08 | |  |
| rs12004669 | 9 | 22365772 | CDKN2B-AS1,DMRTA1 | | 0.12 | | intergenic | | 2.14E-08 | |  |
| rs7030782 | 9 | 22381899 | CDKN2B-AS1,DMRTA1 | | 0.067 | | intergenic | | 2.44E-08 | |  |
| rs496594 | 9 | 22639438 | DMRTA1,LINC01239 | | 0.081 | | intergenic | | 2.00E-08 | |  |
| rs58686769 | 16 | 18930109 | SMG1 | | 0.066 | | intronic | | 0.000000021 | |  |
| rs4795104 | 17 | 34287400 | LYZL6,CCL16 | | 0.064 | | intergenic | | 1.02E-17 | |  |
| rs4796144 | 17 | 34293003 | LYZL6,CCL16 | | 0.064 | | intergenic | | 1.02E-17 | |  |
| rs149197550 | 17 | 34295254 | LYZL6,CCL16 | | 0.064 | | intergenic | | 9.20E-18 | |  |
| rs80329614 | 17 | 34303312 | CCL16 | | 0.14 | | downstream | | 5.85E-28 | |  |
| rs2063979 | 17 | 34303566 | CCL16 | | 0.42 | | UTR3 | | 2.08E-09 | |  |
| rs33995560 | 17 | 34303771 | CCL16 | | 0.11 | | UTR3 | | 2.09E-27 | |  |
| rs150951362 | 17 | 34304264 | CCL16 | | 0.1 | | UTR3 | | 2.05E-20 | |  |
| rs7216969 | 17 | 34305048 | CCL16 | | 0.1 | | intronic | | 2.09E-27 | |  |
| rs11080368 | 17 | 34305071 | CCL16 | | 0.1 | | intronic | | 1.78E-27 | |  |
| rs11080369 | 17 | 34305164 | CCL16 | | 0.1 | | intronic | | 1.78E-27 | |  |
| rs917015 | 17 | 34305525 | CCL16 | | 0.42 | | intronic | | 1.41E-09 | |  |
| rs75236781 | 17 | 34306470 | CCL16 | | 0.061 | | intronic | | 3.94E-20 | |  |
| rs854680 | 17 | 34309051 | CCL16 | | 0.59 | | upstream | | 1.51E-09 | |  |
