# Supplementary material for: Variants in CCL16 are associated with blood plasma and cerebrospinal fluid CCL16 protein levels
Source: BMC Genomics. 2016 Jun 29;17(Suppl 3):437. doi: 10.1186/s12864-016-2788-x (PMC4943476; doi:10.1186/s12864-016-2788-x)
Supplement: Additional file 2: — File contains a table of SNPs significantly associated with CCL16 levels in CSF by meta-analysis. (DOCX 94 kb) [file 12864_2016_2788_MOESM2_ESM.docx]

| **SNP** | **Chromosome** | **Chromosomal position** | | **Proximal Gene(s)** | | **MAF** | | **Predicted Function** | | **Meta-analysis**  **p-value** | |
| --- | --- | --- | --- | --- | --- | --- | --- | --- | --- | --- | --- |
| rs12143270 | 1 | 100328836 | AGL | | 0.025 | | intronic | | 4.27E-09 | |  |
| rs11102493 | 1 | 113088303 | ST7L | | 0.067 | | intronic | | 3.64E-10 | |  |
| rs7538730 | 1 | 113104997 | ST7L | | 0.067 | | intronic | | 3.64E-10 | |  |
| rs72982508 | 1 | 113116129 | ST7L | | 0.064 | | intronic | | 3.75E-10 | |  |
| rs12092350 | 1 | 113123319 | ST7L | | 0.067 | | intronic | | 3.64E-10 | |  |
| rs113717639 | 1 | 113166244 | CAPZA1 | | 0.067 | | intronic | | 3.64E-10 | |  |
| rs13408416 | 2 | 232661068 | COPS7B | | 0.037 | | intronic | | 1.57E-08 | |  |
| rs10185448 | 2 | 232667302 | COPS7B | | 0.038 | | intronic | | 1.57E-08 | |  |
| rs76060308 | 9 | 94489597 | ROR2 | | 0.058 | | intronic | | 1.17E-09 | |  |
| rs78456068 | 10 | 8771630 | LINC00708,LOC101928272 | | 0.059 | | intergenic | | 7.70E-10 | |  |
| rs2191514 | 12 | 42295076 | PDZRN4,GXYLT1 | | 0.12 | | intergenic | | 4.83E-08 | |  |
| rs12306858 | 12 | 42315657 | PDZRN4,GXYLT1 | | 0.13 | | intergenic | | 4.83E-08 | |  |
| rs12301500 | 12 | 42316027 | PDZRN4,GXYLT1 | | 0.13 | | intergenic | | 4.83E-08 | |  |
| rs10880193 | 12 | 42317770 | PDZRN4,GXYLT1 | | 0.13 | | intergenic | | 4.83E-08 | |  |
| rs11181249 | 12 | 42319404 | PDZRN4,GXYLT1 | | 0.13 | | intergenic | | 4.83E-08 | |  |
| rs12313177 | 12 | 42323106 | PDZRN4,GXYLT1 | | 0.13 | | intergenic | | 4.83E-08 | |  |
| rs11181253 | 12 | 42323774 | PDZRN4,GXYLT1 | | 0.13 | | intergenic | | 4.83E-08 | |  |
| rs11181258 | 12 | 42331432 | PDZRN4,GXYLT1 | | 0.13 | | intergenic | | 4.83E-08 | |  |
| rs2159587 | 12 | 42331762 | PDZRN4,GXYLT1 | | 0.15 | | intergenic | | 4.93E-08 | |  |
| rs1541492 | 12 | 42343506 | PDZRN4,GXYLT1 | | 0.14 | | intergenic | | 4.83E-08 | |  |
| rs74725868 | 14 | 98435105 | LINC01550 | | 0.074 | | ncRNA_intronic | | 3.48E-08 | |  |
| rs455378 | 16 | 11968904 | GSPT1 | | NA | | intronic | | 1.87E-08 | |  |
| rs4795104 | 17 | 34287400 | LYZL6,CCL16 | | 0.064 | | intergenic | | 9.63E-16 | |  |
| rs4796144 | 17 | 34293003 | LYZL6,CCL16 | | 0.064 | | intergenic | | 9.63E-16 | |  |
| rs149197550 | 17 | 34295254 | LYZL6,CCL16 | | 0.064 | | intergenic | | 8.32E-16 | |  |
| rs80329614 | 17 | 34303312 | CCL16 | | 0.14 | | downstream | | 1.67E-19 | |  |
| rs33995560 | 17 | 34303771 | CCL16 | | 0.11 | | UTR3 | | 2.94E-19 | |  |
| rs150951362 | 17 | 34304264 | CCL16 | | 0.1 | | UTR3 | | 1.65E-17 | |  |
| rs7216969 | 17 | 34305048 | CCL16 | | 0.1 | | intronic | | 2.94E-19 | |  |
| rs11080368 | 17 | 34305071 | CCL16 | | 0.1 | | intronic | | 2.32E-19 | |  |
| rs11080369 | 17 | 34305164 | CCL16 | | 0.1 | | intronic | | 2.32E-19 | |  |
| rs75236781 | 17 | 34306470 | CCL16 | | 0.061 | | intronic | | 2.96E-17 | |  |
| rs147220252 | 18 | 69849192 | LOC102724913,CBLN2 | | 0.0078 | | intergenic | | 5.34E-08 | |  |
| rs8109739 | 19 | 53043126 | ZNF808 | | 0.068 | | intronic | | 9.22E-09 | |  |
