# Supplementary figures and images for: Variants in CCL16 are associated with blood plasma and cerebrospinal fluid CCL16 protein levels
Source: BMC Genomics. 2016 Jun 29;17(Suppl 3):437. doi: 10.1186/s12864-016-2788-x (PMC4943476; doi:10.1186/s12864-016-2788-x)

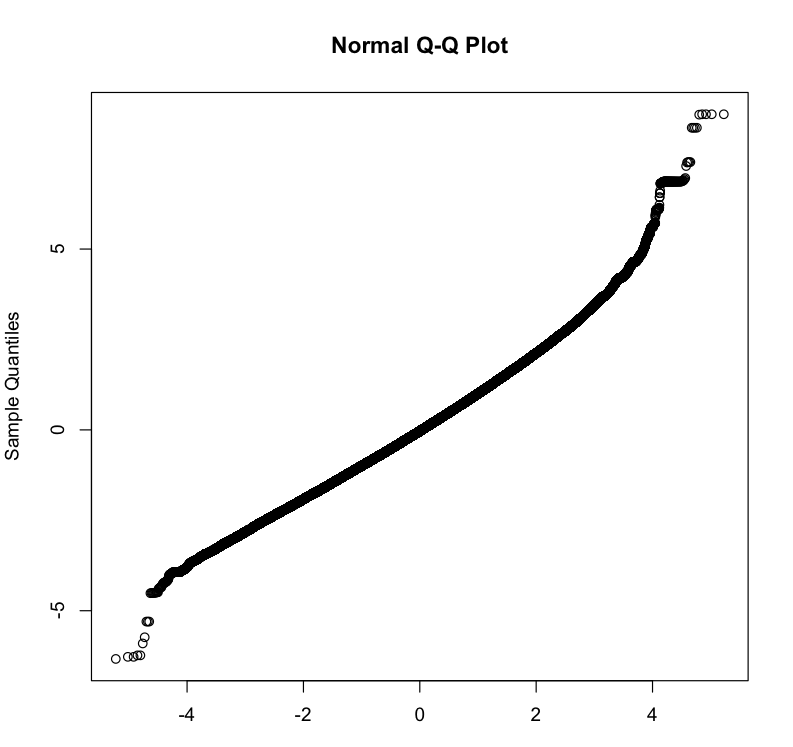

Supplement: Additional file 3: — File contains a Q-Q plot of the CSF data used in this study. (DOCX 82 kb) [file 12864_2016_2788_MOESM3_ESM.docx]

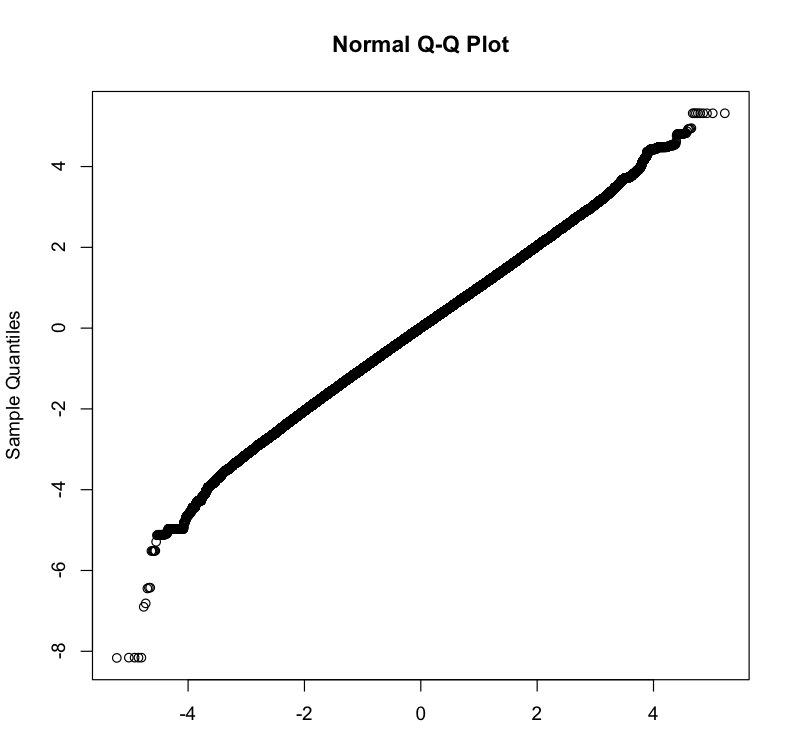

Supplement: Additional file 4: — File contains a Q-Q plot of the plasma data used in this study. (DOCX 83 kb) [file 12864_2016_2788_MOESM4_ESM.docx]
